# ARIMA model forecasting analysis of the prices of multiple vegetables under the impact of the COVID-19

**DOI:** 10.1371/journal.pone.0271594

**Published:** 2022-07-28

**Authors:** Lisha Mao, Yin Huang, Xiaofan Zhang, Sijin Li, Xiangni Huang

**Affiliations:** School of Logistics and Transportation, Central South University of Forestry and Technology, Changsha, Hunan, China; Vellore Institute of Technology: VIT University, INDIA

## Abstract

As a large agricultural country, China’s vegetable prices affect the increase in production and income of farmers and the daily life of urban and rural residents and influence the healthy development of Chinese agriculture. 51,567 vegetable price data of 2020 are analyzed to determine the factors that influence vegetable price fluctuations in two dimensions (vertical and horizontal) in the special context of the COVID-19, and an ARIMA model of short-term price prediction is then employed and evaluated. Based on the factors affecting vegetable prices, the results of the model are further examined. Finally, pertinent suggestions are made for the development of the local vegetable industry in the post-epidemic era.

## Introduction

The COVID-19 outbreak in 2020 has attracted global attention. Various strata of society has been affected to a certain extent. Many scholars have invested in research related to COVID-19 [1–3]. In China, the outbreak of COVID-19 has caused a nationwide Level-I response to a major public health emergency, which severely affects China’s harmonious socio-economic order, and inevitably harms both rural economy and vegetable supply chain which are prone to be affected by various factors in China. Vegetable prices can directly influence the income of farmers and the living standards of residents, which in turn affects the development of China’s vegetable industry and the overall balance of the national economy. As a necessary material for residents’ daily lives, the stability of vegetable prices plays an essential role in the prevention and control of the epidemic and social stability during the COVID-19 pandemic; however, in recent years, vegetable prices have fluctuated frequently, and this has become a major challenge for China’s vegetable industry.

Although China’s vegetable industry has developed rapidly, this has occurred mainly in terms of quantity and scale, and a process that allows the vegetable industry to change from the low-end stage that attaches importance to quantity to the high-quality development stage is required [4]. The outbreak of COVID-19 in 2020 has affected all industries in China, but it is also an opportunity for the vegetable industry to transform into one embodying the precepts of modern agriculture. Many scholars have invested in China’s vegetable industry under COVID-19: Dong has discussed the effects of COVID-19 on China’s agricultural economy and mitigation measures [5]; Wu studied the strategy of vegetable supply during implementation of COVID-19 epidemic prevention measures [6]; Li [7] and Ren [8] explored the current situation and countermeasures applicable to the vegetable supply chain in Wuhan and the Chaoyang District of Beijing through the epidemic; however, these studies propose only macro-scale countermeasures and suggestions for the current situation and development of the circulation of agricultural products and vegetables. Few scholars have analyzed China’s vegetable prices in time and space under the background of the epidemic in 2020 based on actual big data pertaining to vegetables. Therefore, in the present research, a comprehensive analysis is undertaken from both horizontal and vertical perspectives by using big data analysis and mining technology and data geographic visualization techniques.

Today, with the rapid development of Internet technology, big data analytics (BDA) has attracted increasing attention: research on “big data plus agriculture” is also increasing. Shen used big data technology to study the intelligent supply chain network model of agricultural products [9]. Zhu evaluated the advantages of big data sharing based on blockchain, and established a big data sharing model based on blockchain [10]. Viet studied the expected delivery of agricultural products under the background of agricultural food supply chain based on data driving [11]. It can be seen that in practice, the innovative integration of big data and agricultural price forecasting has become an important path via which to promote agricultural development.

The use of big data technology, mining and visualization techniques to analyze and mine vegetable data and make intelligent forecasts of vegetable prices through big data modeling can help farmers, enterprises and the government to assess the risks in the supply chain of vegetable production and sales, which is of practical significance in guiding farmers to adjust their production, avoiding market risks, as well as ensuring the orderly operation of the agricultural market. In recent years, many scholars have performed a series of exploratory analyses and empirical studies on price forecasting of agricultural products, such as soybean [12], pork [13, 14], beef [15], and corn [16] price forecasting problems. People are increasingly concerned about how to reduce or even avoid the economic losses caused by price fluctuations, which has prompted many scholars to engage in the study of agricultural futures price forecasting, such as Wu *et al*. [17] (SVJ), He and Wen [18] (time-frequency analysis), and Wang *et al*. [19] (radial basis function neural network). Among them, since the price fluctuations of most agricultural products show more obvious time series characteristics, numerous scholars use time series models to predict agricultural prices. For example, Zhao [20] predicted agricultural futures prices based on machine learning algorithms, and Dipankar [21] used a long-memory time series model to predict the price of rice in India.

In terms of agricultural price research, most of the research is focused on the macroscopic trends in agricultural price analysis and forecasting, or on the problem of price forecasting for a single agricultural product; few scholars have studied and forecast the prices of multiple vegetables. In summary, it is is imperative to perform data-driven analysis and forecasting of vegetable prices in the context of the COVID-19.

To evaluate the price fluctuations of vegetables under the influence of the COVID-19 pandemic in 2020, a web-crawling technique is adopted to crawl price data of three different types of vegetables, namely leafy vegetables, root vegetables, and solanaceous fruits vegetables in 2020, on the Chinese Vegetable net (http://www.vegnet.com.cn/) and an ARIMA model is fitted to undertake an intelligent forecast of the prices of the three types of vegetable based on Python programming.

## Data and methods

### Data source

**Fig 1:**
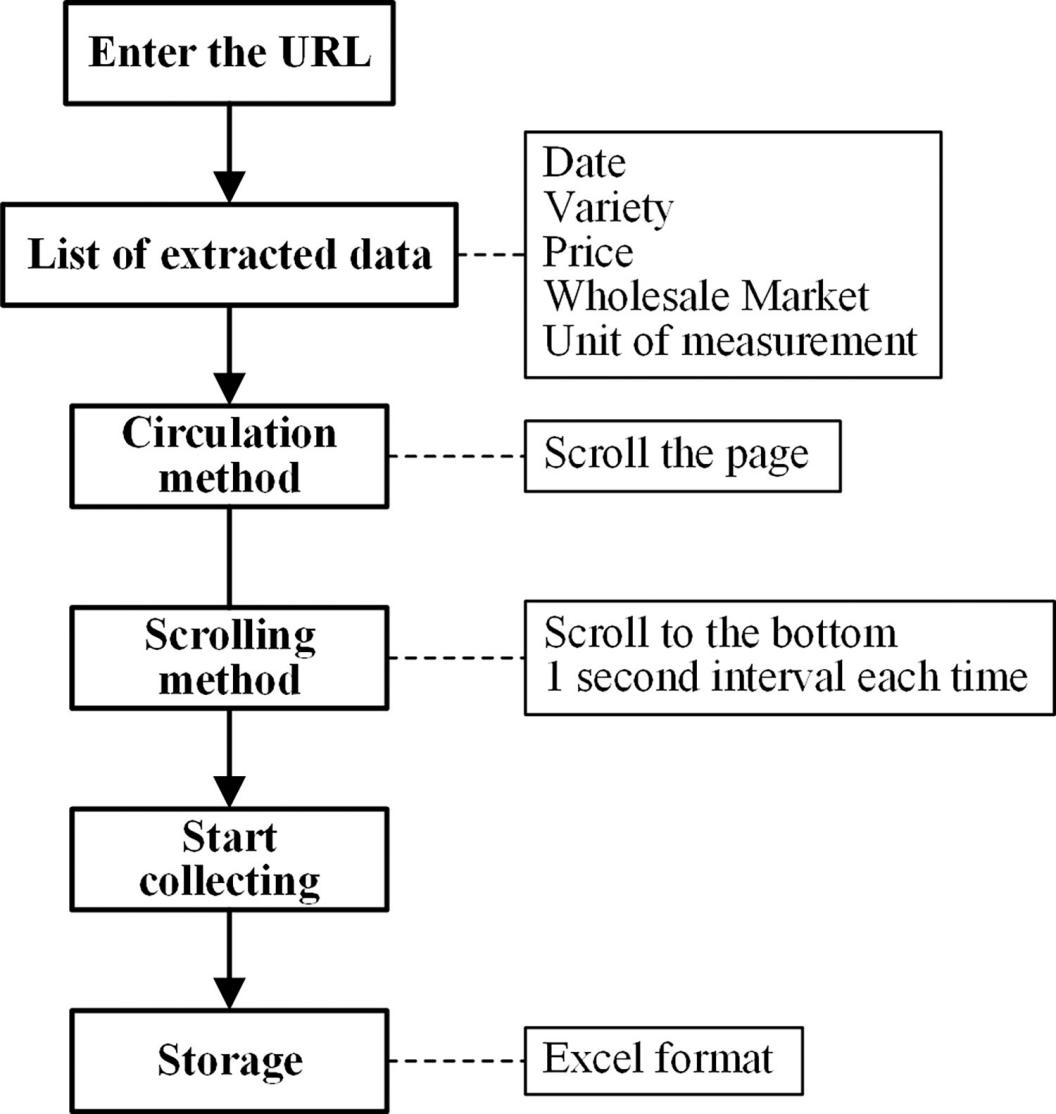
Octopus data collection process.

As shown in Fig 1, the Octopus collector is used to crawl the vegetable wholesale price data on the vegetable website (http://www.vegnet.com.cn/) from January 1, 2020 to November 19, 2020. For each of the three vegetable categories on the website, a typical vegetable is selected as the representative of this type, namely cabbage (leafy vegetables), carrot (root vegetables), and solanaceous fruit vegetables (eggplant).

First, we find the website address of each vegetable selected in the Vegetable Net at the corresponding time. For example, on the Vegetable Net, the price data of Chinese cabbage in 2020 covers the data from January 1 to November 19, and the Vegetable Net is recorded in reverse order. Therefore, we enter the website for November 19, 2020 into the Octopus interface, turn back and crawl the data to January 1, 2020: in the Vegetable Net page in the Octopus collector, the dimension required by the data is selected to form the content of the data list. Afterwards, we click PageDown and select the “next page” button in the page to set the automatic page turning program. The scrolling mode is set to “scroll to the bottom”, so that the page number can be checked at any time during the automatic crawling process, so as to understand the data crawling progress. Finally, a total of 51,567 items of data are obtained, with data dimensions including date, vegetable variety, wholesale market, minimum price, maximum price, average price, and unit of measurement. The data collection process is illustrated in Fig 1.

The omission method is used based on the eight-two ratio division, to select price data from January to September in 2020 as the training set for fitting the ARIMA model, while the price data from October and November are used as the test set to assess the predictive effect of the model.

### Research methodology

#### (1) Big data analysis and mining techniques

The research is performed based on big data analysis and mining techniques, and uses methods such as Time-Series Analysis to reveal deeper reasons that cannot be derived from observational charts. Big data analysis, mining and visualization techniques are integrated to explore the trends in three vegetable-price curves and their causes, based on longitudinal and cross-sectional perspectives.

Data-geographic visualization technology is to visualize the data or the results of data analysis on the map to help understand the trends (if any) in data. Among them, in the horizontal perspective, the data-geographic visualization technique is used to represent the price with the color depth, analyze the distribution of the national vegetable price, and mine its influencing factors according to the distribution characteristics of vegetable price.

#### (2) Intelligent prediction of big data based on machine learning

Machine learning is the core of artificial intelligence technology, and time series refers to a series of values of the same statistical indicators arranged in chronological order. The main purpose of time series analysis is to make predictions about the future based on existing historical data. Herein, a classical algorithm in machine learning, the time series ARIMA model, is used to fit price data for three different types of vegetables and the vegetable prices are forecasted using the ARIMA model after parameter fitting.

ARIMA, as a time series forecasting method, can be employed to understand the data through time series analysis and study the sequence formed by the state of the variables at different times. It can also be used to fit the data and make forecasts, quantitatively describing the pattern of variables in the time series and future trends.

The advantage of the ARIMA model over other forecasting models is that it only requires forecasting based on endogenous variables, without the need to acquire other relevant exogenous variables, and focuses more on the patterns and trends of the variables themselves to be studied. The endogenous variables are those that are explained or predicted in the model, while the exogenous variables only affect the system and are not affected by the system.

**Fig 2:**
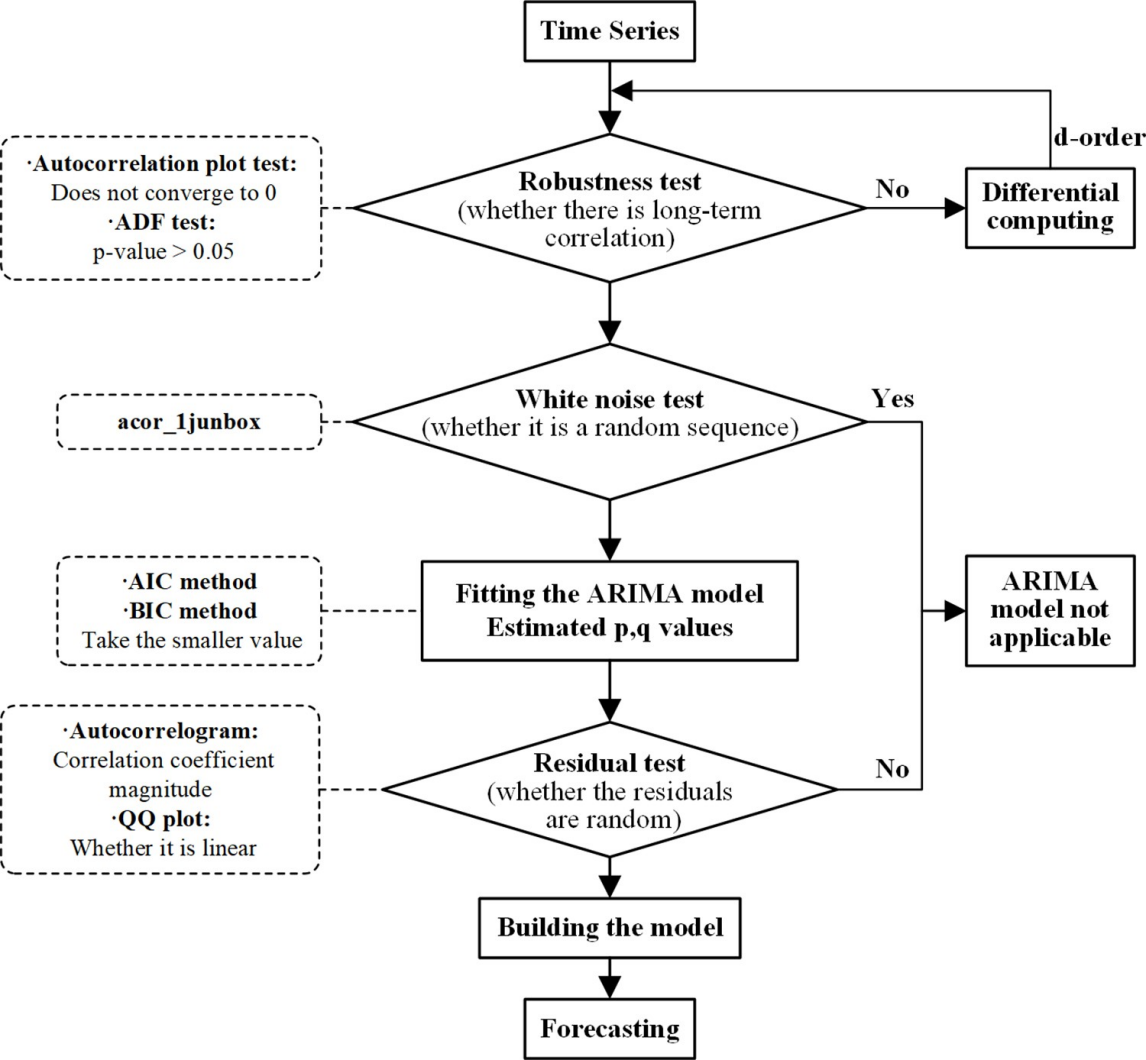
ARIMA model flowchart.

The flowchart through the ARIMA model used herein is shown in Fig 2.

## Results

### Analysis of price series fluctuations of multiple vegetables based on data analysis and mining

#### (1) Vertical analysis of price series of multiple vegetables

Three specific time points are selected to classify the epidemic phases according to the COVID-19 dynamics published on the website of the Health and Welfare Commission: February 18, 2020—the number of new cured patients exceeds the number of new confirmed cases nationwide, March 18, 2020—the first time there are no new confirmed cases nationwide, and May 22, 2020—the first time there are zero new cases nationwide. The year 2020 can be divided into an outbreak phase (January 1- February17, 2020), an epidemic mitigation phase (February 18 -March 17, 2020), an epidemic interruption phase (March 18—May 21, 2020), and the normalization phase (May 22—November 19, 2020). The price data for each of the three vegetables are analyzed and mined in the context of the evolution of the epidemic in China in 2020.

**Fig 3:**
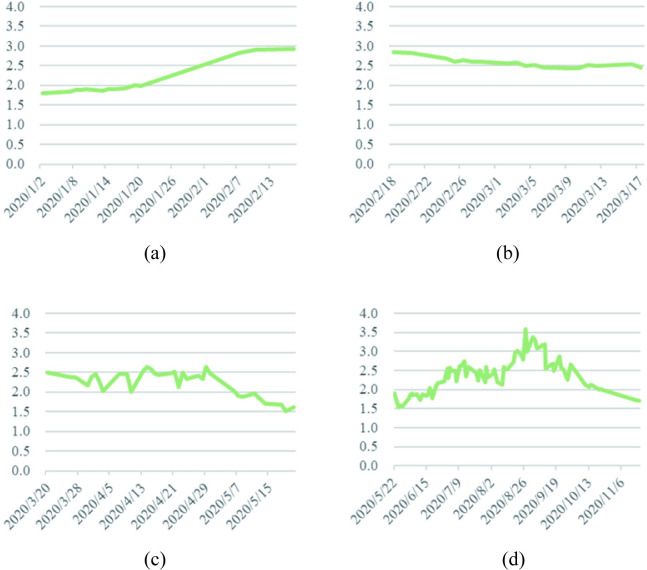
Cabbage prices in 2020. (a) During the outbreak phase. (b) During the epidemic mitigation phase. (c) During the epidemic interruption phase. (d) During the normalization phase of epidemic prevention.

*a*. *Analysis of cabbage price series*. The fluctuation of cabbage prices in 2020 over the four phases is illustrated in Fig 3.

During the outbreak phase (January 1—February 17, 2020), the price curve of cabbage showed a continuous increase, from 1.81 RMB/kg initially to 2.93 RMB/kg. On the one hand, the price of cabbage was relatively low due to the fact that it matured in winter and there was an adequate supply of cabbage at the beginning of the year; on the other hand, owing to the sudden outbreak of the disease, the supply of vegetables gradually tended to be in short supply as the government restricted travel among residents, gatherings and the opening of various places, and therefore consumers’ frantic buying behavior for commodities led to a continuous increase in the price of cabbage at the beginning of the outbreak. On January 20, 2020, the National Health Commission issued Announcement No. 1 to include the COVID-19 in *the Law of the People’s Republic of China on the Prevention and Control of Infectious Diseases*, which attracted the attention of the whole country. As a result, residents’ panic about the shortage of supplies caused the price curve of cabbage, a household vegetable, to rise sharply since January 20, 2020.

With the strengthening of national measures to prevent and control COVID-19, the number of new patients cured exceeded the number of new confirmed cases on February 18, 2020, entering the epidemic mitigation phase. As China began to push for the resumption of work and production while the COVID-19 was being prevented and controlled, the nation’s fears of a shortage of supplies gradually eased, so cabbage prices fell during the epidemic mitigation phase. However, as the first wave of the massive epidemic, mainly in Wuhan, was not fully contained during the epidemic mitigation phase, the impact of the COVID-19 on the price of common vegetables such as cabbage always existed and therefore the price of cabbage was slow to rebound.

On March 18, 2020, for the first time in China, there were no new confirmed local cases and the country entered the epidemic interruption phase. Wuhan’s various cabin hospitals were adjourned, and the first batch of 49 national medical teams aided by Wuhan were evacuated from the city, by which time the spread of the local epidemic in China, with Wuhan as the main battleground, had been largely blocked. In addition, policy and technical support during the spring farming season was increased and the supply of agricultural production was increased to promote spring agricultural production in a precise and orderly manner. Consequently, cabbage prices trended downward in this stage under the influences of the epidemic easing and macro-control.

On May 22, 2020, the number of new cases nationwide was 0 for the first time, while China’s overall epidemic prevention and control entered a phase of normalization. Cabbage prices reached their lowest point since the COVID-19 in these days, even falling below the price at the beginning of the year. On June 16, 2020, the outbreak of a home-grown epidemic in Beijing increased the level of response to a public health emergency to Level 2, and cabbage prices saw their first small peak in the epidemic prevention normalization phase, with prices reaching 1.87 RMB/kg. Due to the high summer rains and temperatures, which were not conducive to cabbage production and planting, and the high temperatures making it difficult to store leafy vegetables, the prices of cabbage increased again and reached 3.59 RMB/kg in August/September, making the price of cabbage the highest in 2020. At this stage the domestic epidemic was generally sporadic, but through a series of effective prevention and control measures, the government were able to intercept the transmission chain at the first sign of an epidemic, and the impact of the COVID-19 on vegetable prices gradually diminished, so cabbage prices fell to 1.71 RMB/kg at the end of the year.

**Fig 4:**
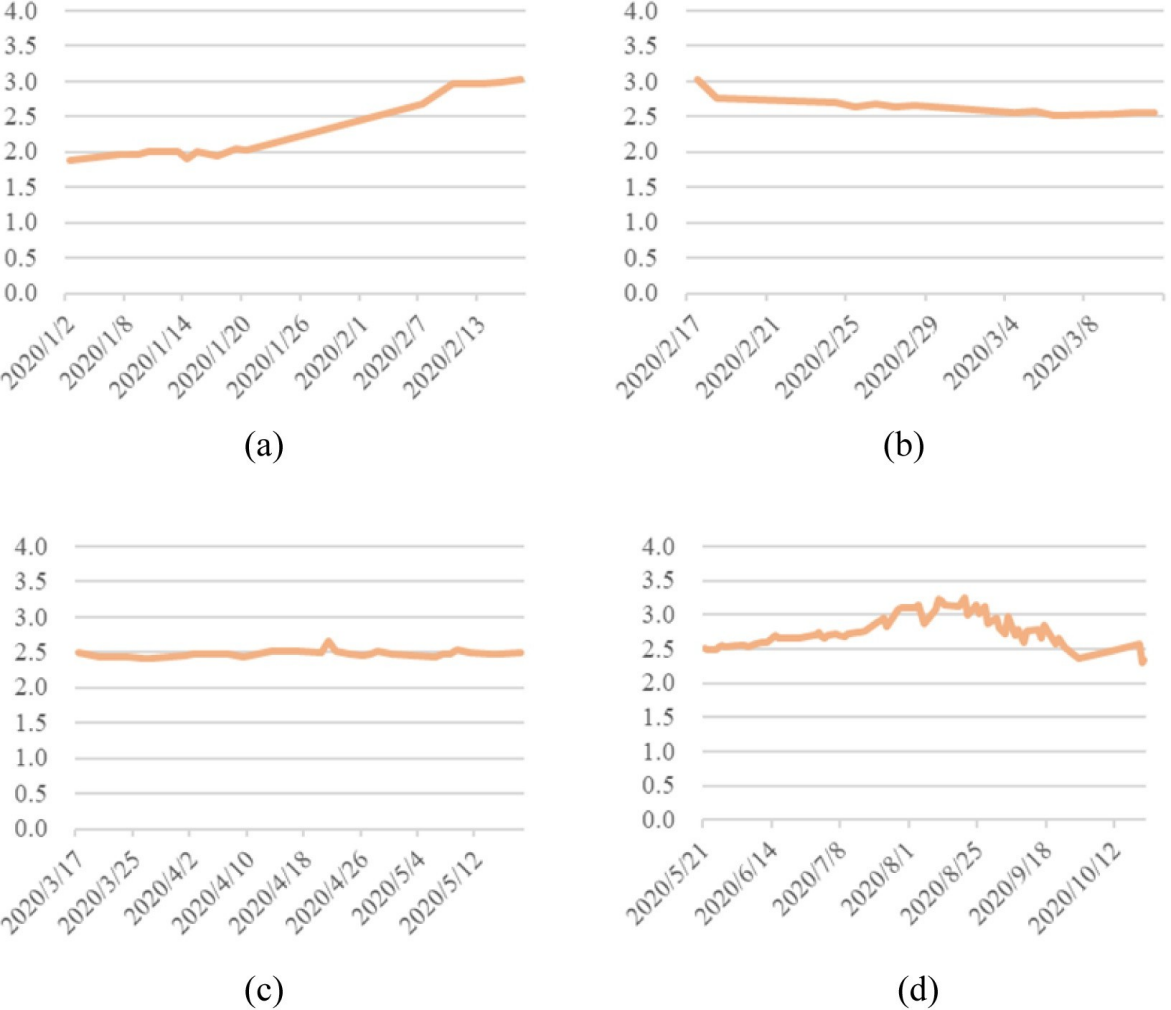
Carrot prices in 2020. (a) During the outbreak phase. (b) During the epidemic mitigation phase. (c) During the epidemic interruption phase. (d) During the normalization phase of epidemic prevention.

*b*. *Analysis of carrot price series*. The fluctuations in carrot prices over the four stages in 2020 are illustrated in Fig 4.

As cabbage and carrots are both winter household vegetables, the price trends of cabbage and carrots were highly similar over the same time period (January 1, 2020 -November 19, 2020) under the impact of the COVID-19. During the outbreak phase (January 1—February 17, 2020), the strong epidemic shock caused the price curve for carrots to continue to rise, from 1.89 RMB/kg to 3 RMB/kg; during the epidemic mitigation phase (18 February—17 March 2020), prices fell back as the epidemic was contained and thus less influential on prices, falling to 2.5 RMB/kg; while during the epidemic interruption phase (18 March—21 May 2020), carrot prices tended to stabilize at the post-decline level with no obvious trend because of the continued impact of the epidemic; carrot prices rose and then fell, and its fluctuation is relatively obvious during the normalization phase of epidemic prevention.

Fresh vegetables are generally more volatile in price due to their perishability, carrots, as a root vegetable, are less perishable and more resistant to storage than other types of fresh vegetables. Therefore, when comparing the price curves of the three vegetables, the price curve of carrots was relatively stable, *i*.*e*. the price movements of root vegetables were less frequent than those of leafy vegetables and eggplant vegetables. The carrot prices particularly showed an evident stability during the epidemic mitigation phase and the epidemic interruption phase, as there were no sudden shocks from the epidemic and no adverse effects from the high summer temperatures and rain.

During the normalization phase of disease prevention (May 22—November 19, 2020), carrot prices grew and then fell, reaching a peak price of 3.25 RMB/kg in the summer in 2020. This is determined by the growth habits of carrots, which require a hot environment during the seedling stage and often a cooler temperature environment when the roots are fat, so carrots are usually sown in summer and autumn, harvested and marketed for sale in autumn and winter. Therefore, even though the epidemic has largely stabilized during the normalization phase of epidemic prevention and the epidemic exerts less influence on vegetable prices, the counter-seasonal nature of carrots still makes carrot prices higher in the summer.

**Fig 5:**
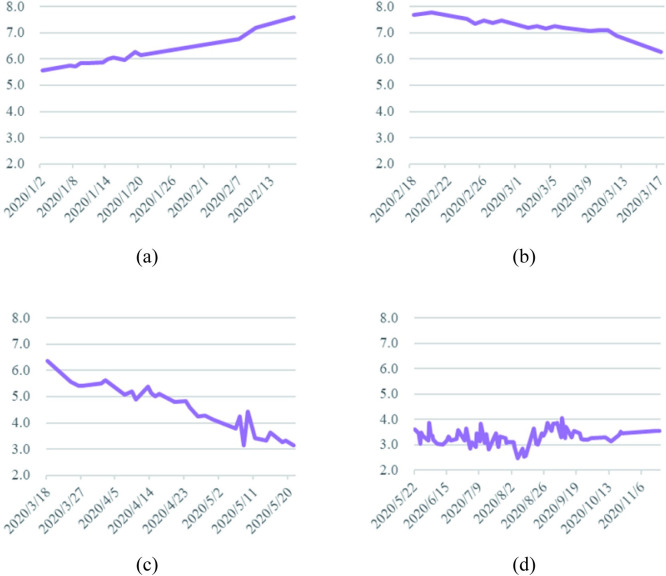
Eggplant prices in 2020. (a) During the outbreak phase. (b) During the epidemic mitigation phase. (c) During the epidemic interruption phase. (d) During the normalization phase of epidemic prevention.

*c*. *Analysis of eggplant price series*. The fluctuations of eggplant prices in four stages in 2020 are displayed in Fig 5. Eggplants are more expensive overall than cabbages and carrots due to the more selective production conditions and the need to choose sandy loamy soils with high terrain for cultivation.

During the outbreak phase (January 1—February 17, 2020), the price of eggplant continued to rise from 5.56 RMB/kg to 7.59 RMB/kg, which was mainly influenced by the sales link of supply chain. The counter-seasonal nature of eggplant, the phenomenon of holiday price increases and the COVID-19 all affected the sales price of eggplant. As eggplant is a seasonal vegetable (most available in summer), the price of counter-seasonal eggplant was higher in winter, and the outbreak of the epidemic at the beginning of the year led to a 36% increase in the price of eggplant from January 1 to February 17, 2020. On January 20, 2020, Zhong Nanshan and other experts informed the media that COVID-19 could "spread from person to person", which caused a nationwide sensation. Therefore, the price of eggplant rose sharply after a small peak on January 20, 2020.

The price of eggplant began to fall, from 7.67 RMB/kg to 6.26 RMB/kg, as the government took COVID-19 more seriously and implemented a series of effective measures to prevent the epidemic, thus weakening the impact thereof on the vegetable market. However, due to a small rebound in the epidemic, the rate of price reduction (5.03%) was not as fast as the price increase (13.53%) during the outbreak phase.

The epidemic situation continued to improve as the epidemic entered a blocking phase on March 18, 2020, with no new confirmed indigenous cases in the country for the first time, and eggplant prices remained low. March 25, 2020 saw the country enter a period of concentrated rainfall, with a prolonged and concentrated rainy season unfavorable to the growth of eggplant. The mid-April to early summer period, however, saw a period of drought in many parts of the country, which adversely affected the growth of eggplant. Thus, prices of eggplant decreased below the beginning of the year at the beginning of April, then continued to fall, reaching 3.14 RMB/kg on May 22, 2020, the lowest price since the beginning of the year.

During the normalization phase of epidemic prevention (May 22—November 19, 2020), the country entered the rainy season one after another on May 29, 2020, with the duration and amount of rain hitting a record high since 1961, and eggplant, as a perishable vegetable, was subjected to high temperatures over a long summer, with rapid deterioration and high losses in storage and transportation, so the price of eggplant was always low during the normalization phase of epidemic prevention. In addition, although domestic epidemic prevention methods have continued to mature and are able to isolate the spread of the epidemic in the first instance, the epidemic has always rebounded on a small local scale, for example, the outbreak of a local epidemic in the Xinfadi wholesale market in Beijing on June 11 and the epidemic in Qingdao on September 24, leading to very significant fluctuations in the price of eggplant.

#### (2) Horizontal analysis of price series of multiple vegetables

To study the effects of the epidemic on the national vegetable prices, this study additionally selects the vegetable price data pertaining to February 2019 to compare with that in February 2020, uses Echarts to conduct data geographic visualization of the national vegetable price data at the selected time, and analyzes the influences of the spread of COVID-19 and other factors on the vegetable supply chain from a horizontal perspective.

**Fig 6:**
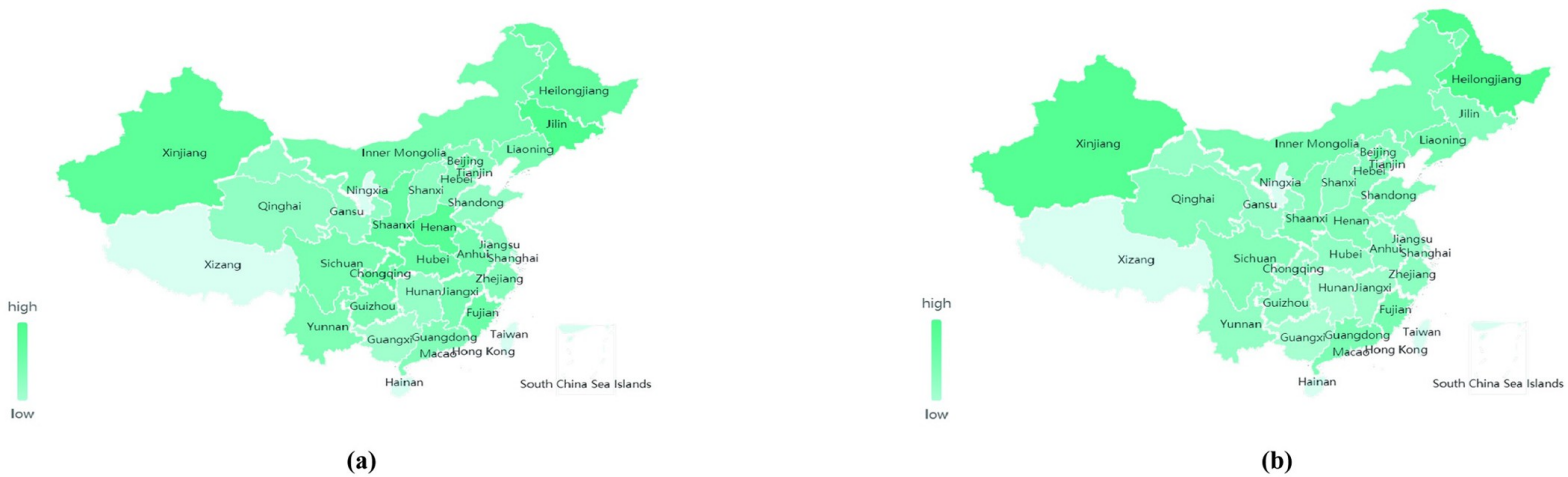
February 2019 (a) and February 2020 (b) National cabbage price map.

*a*. *Cabbage data-geographic visualization analysis*. Taking the data of Chinese cabbage as an example, the geographical distribution of the price of leafy vegetables is analyzed by data geographical visualization (Fig 6).

As shown in Fig 6, the regions with the highest price of cabbage in February 2020 are Xinjiang and Heilongjiang, which are the vegetable supply bases in summer due to their high latitude. As an autumn or winter vegetable, Chinese cabbage mainly depends on the “southern vegetable—northern transportation route” to supply Xinjiang, Heilongjiang and other regions. After the COVID-19, due to strict traffic control, long-distance vegetable transportation was affected, resulting in a shortage of cabbage in these two regions and the continuous rise of cabbage prices, making them the regions with the highest price of Chinese cabbage.

As can be seen from Fig 6, after the epidemic in February 2020, the price distribution of Chinese cabbage across the country has changed significantly, and the price levels of the Huang Huai Hai region, the Bohai Rim region, the Yangtze River region, and the south-west region have gradually become consistent. This is because the temperature in these three regions is suitable for the growth of Chinese cabbage after the epidemic, and the supply of Chinese cabbage is relatively stable. In addition, the three production areas are less affected by the epidemic, therefore, the fluctuation range of cabbage price therein under the COVID-19 is relatively small.

**Fig 7:**
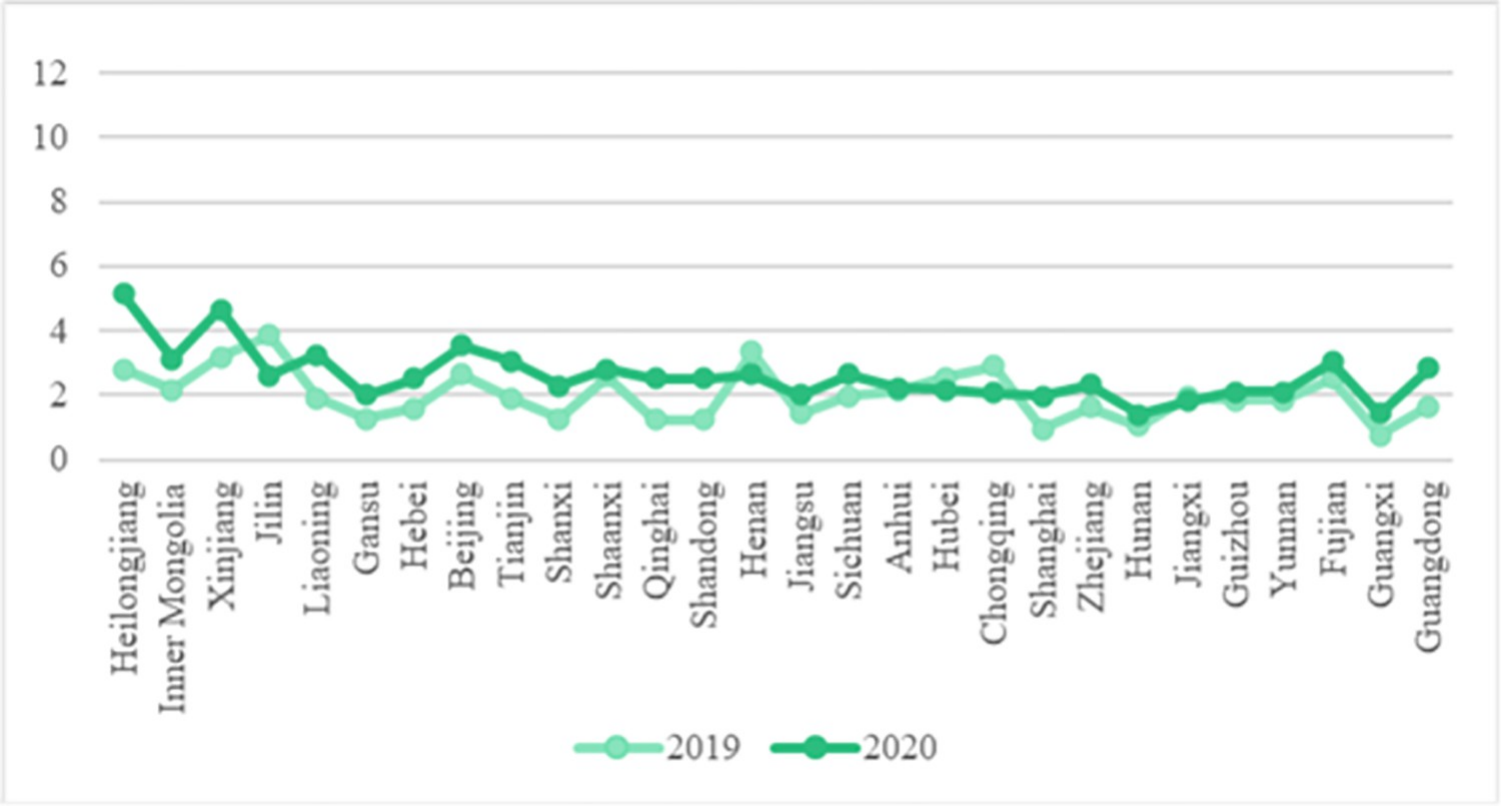
Broken line chart of cabbage price comparison across the country.

Compared with the national price of Chinese cabbage in February 2019, the price of Chinese cabbage increased to varying degrees after the epidemic in February 2020 (Fig 7). Among them, the price of cabbage in Heilongjiang increased the most. Compared with the price of Chinese cabbage before the epidemic (*e*.g. in February 2019), the price of Heilongjiang Chinese cabbage increased by 2.30 RMB/kg during the epidemic in February 2020. This is because Heilongjiang lies within the summer and autumn vegetable supply base. Its production environment, climate, and planting structure are not conducive to the production of winter vegetables such as cabbage. Its price itself is easily affected by market supply and fluctuates. Affected by the spread of the epidemic, the supply of Chinese cabbage in Heilongjiang was blocked, and its price rose the most.

**Fig 8:**
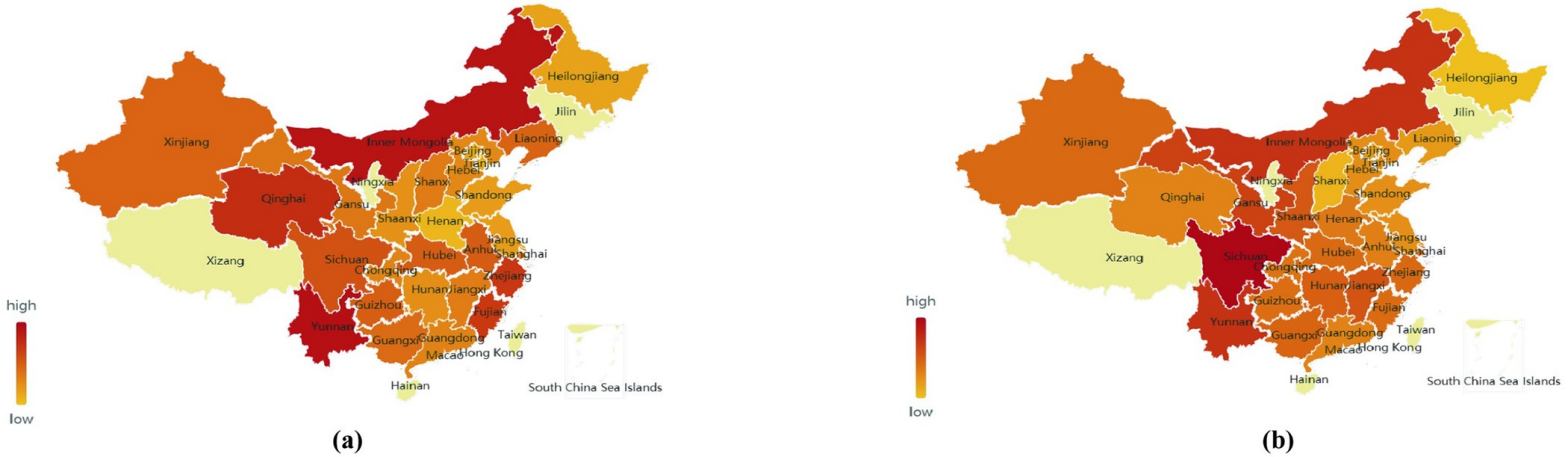
February 2019 (a) and February 2020 (b) National carrot price map.

*b*. *Carrot data-geographic visualization analysis*. Taking carrot data as an example, the data-geographic visualization analysis of root vegetables supply chain is conducted (Fig 8).

After the epidemic in February 2020, based on the national price level, the price of carrots in the north of the Qinhuai front line generally decreased, while the price in the south of Qinhuai generally increased (Fig 8). This is because carrots are summer vegetables, while most of the areas in the north of Qinhuai River are summer and autumn vegetable supply bases. In the case of restricted traffic, this further amplifies the advantage of sufficient carrot supply in the North. Therefore, the price of carrots in the North tends to be stable under the influence of the epidemic.

**Fig 9:**
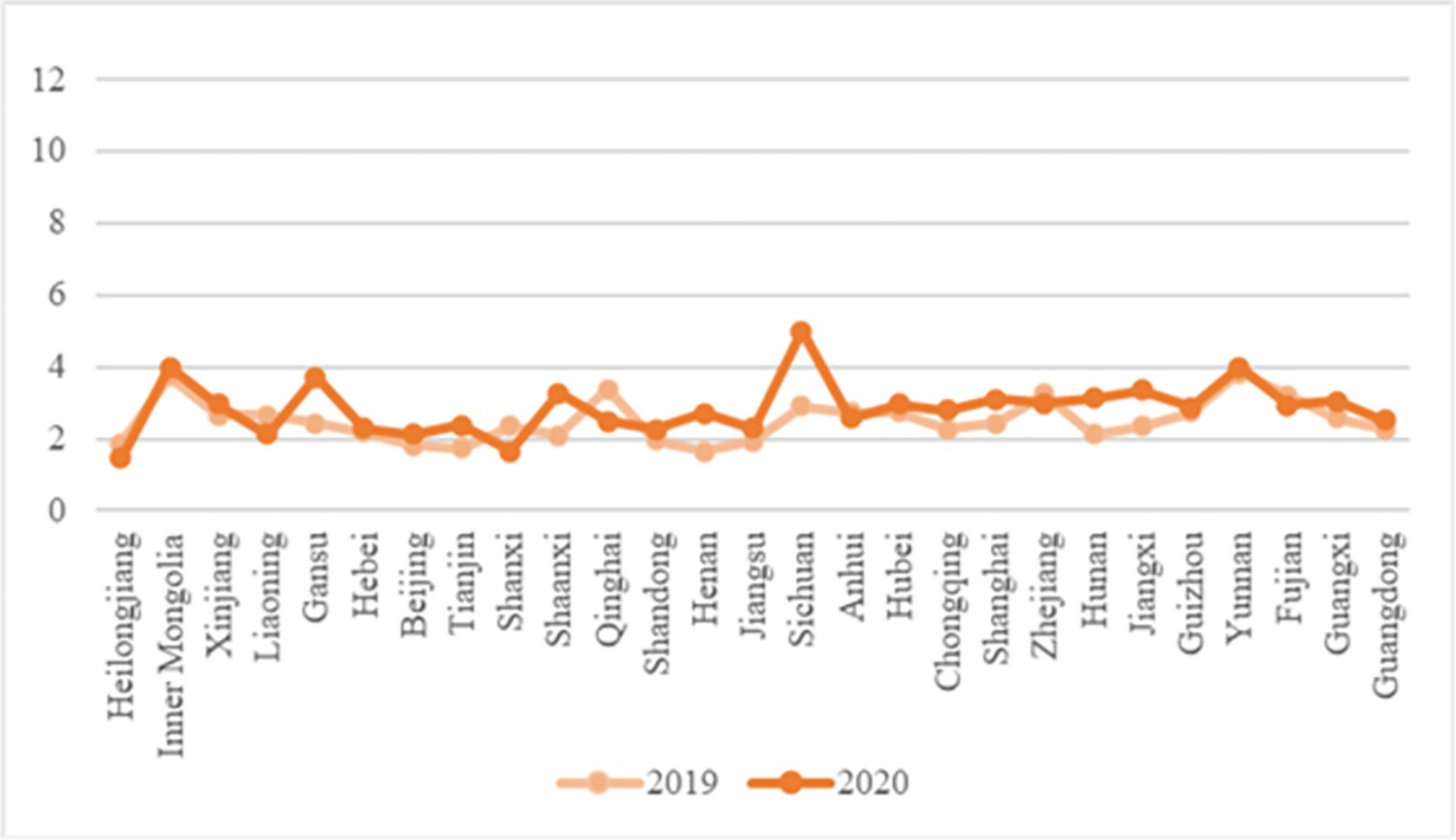
Broken line chart of carrot price comparison across the country.

As shown in Fig 9, after the COVID-19 outbreak in February 2020, the rising trend in carrot prices was particularly obvious in Gansu and Sichuan Province, rising by 2.10 RMB/kg and 1.28 RMB/kg respectively. In other parts of China, the fluctuation in carrot price is less than 1 RMB/kg. Gansu has a long and narrow terrain and comprises an extremely complex landform. Sichuan Province spans several major terrain units such as plateau, mountain, and basin landforms. Although the price of carrots is generally stable under the epidemic, it is greatly affected by the complex terrain, and the haulage of vegetables in some areas is hindered. This shows that the price of carrots is affected by traffic controls introduced under the epidemic, and the phenomenon of traffic obstruction is more serious, especially in areas with complex terrain such as plateau and basin landforms. Due to the lack of transportation routes, the phenomenon of insufficient supply caused by transportation is more serious. In addition, as a root vegetable, carrots are much less perishable, their price is relatively stable compared with other vegetables, and its price fluctuation is less affected by the epidemic.

**Fig 10:**
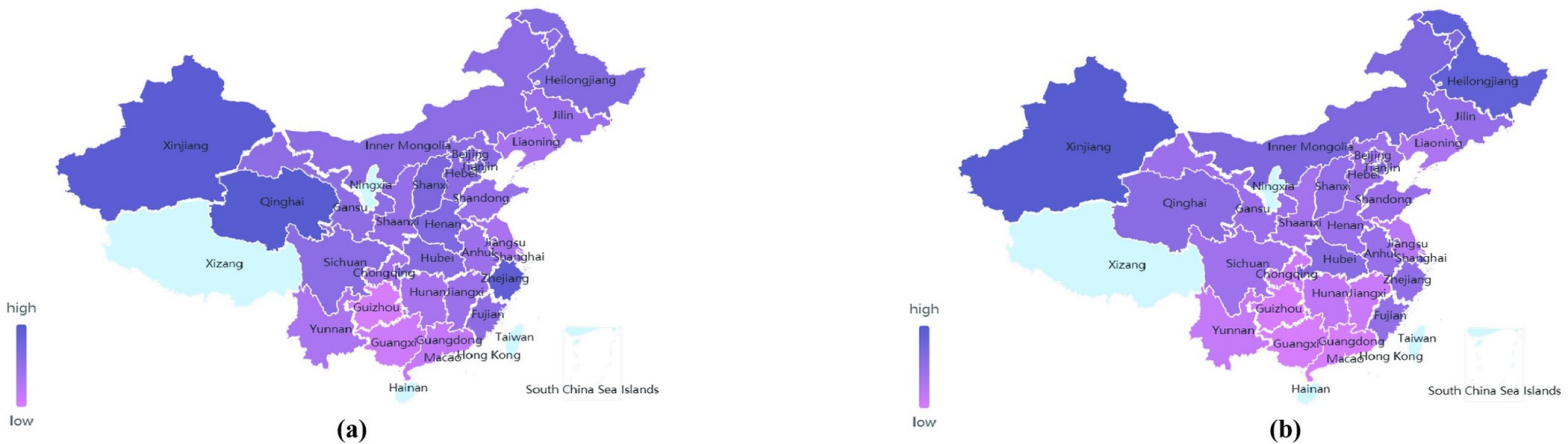
National eggplant price map in February 2019 (a) and February 2020 (b).

*c*. *Eggplant data-geographic visualization analysis*. Taking eggplant data as an example, the data-geographic visualization analysis of eggplant fruit vegetable supply chain is conducted (Fig 10).

As shown in Fig 10, after the February 2020 COVID-19, the price of eggplant in the northeast and North China has increased relative to the national average price. Compared with the national average price, the price level of eggplant in other regions has decreased. The northeast and North China are the main supply bases of summer and autumn vegetables, while the supply of eggplant (as a winter vegetable) is relatively insufficient. The growth of eggplant likes water and is afraid of waterlogging, while the areas in the north of Qinhuai River are mostly dry, which is not conducive to the growth of eggplant. Therefore, after the outbreak, eggplant production in Northeast and North China was hindered by the comprehensive influence of production environment, precipitation, planting structure, and other factors. Meanwhile, under the impact of the epidemic, traffic control has blocked the transportation link of supply chain, and the insufficient supply of eggplant in Northeast and North China has led to a significant increase in its price.

**Fig 11:**
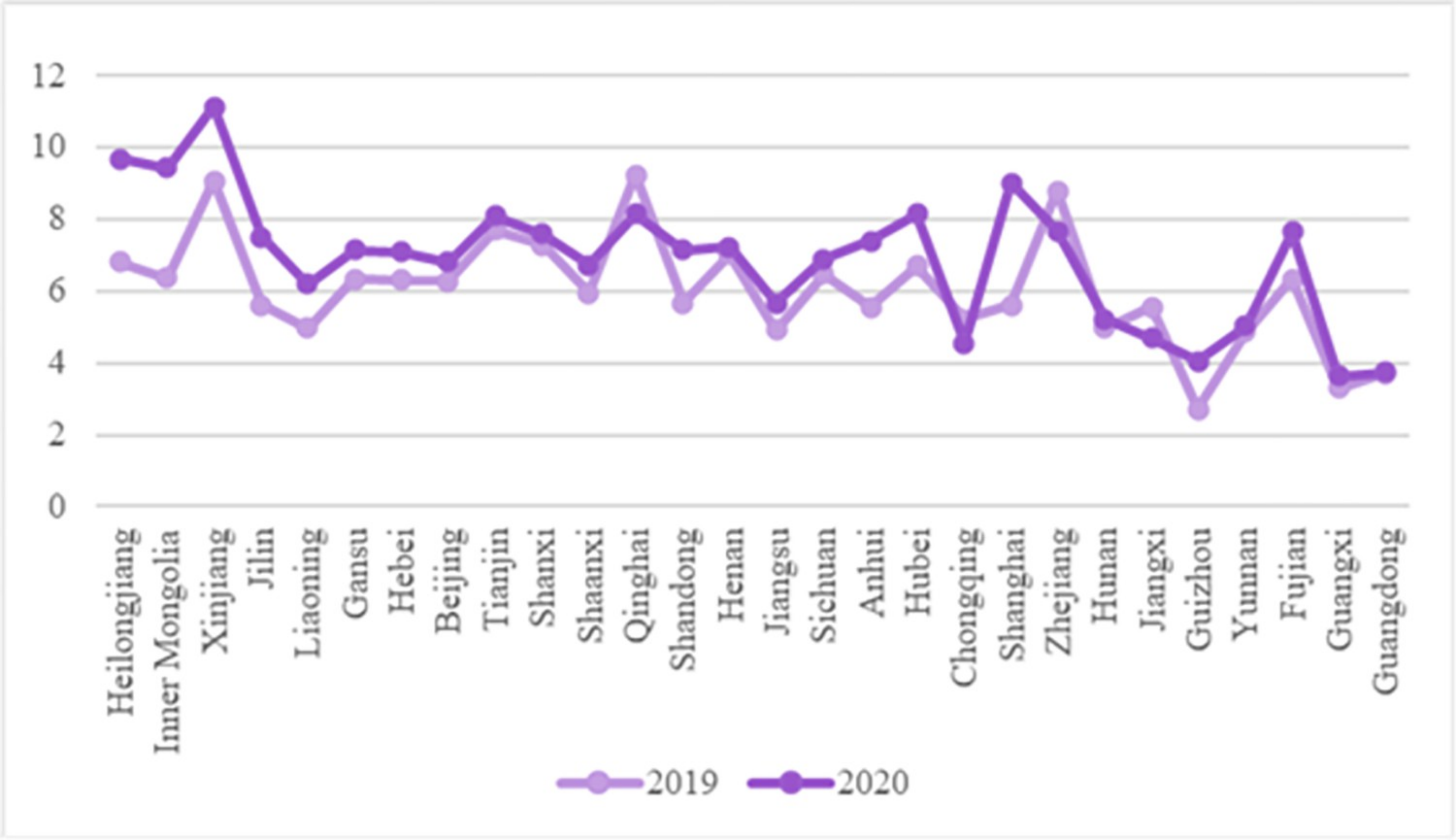
Broken line chart of eggplant price comparison across the country.

As shown in Fig 11, the price of eggplant fluctuates significantly under the impact of the epidemic, indicating that the prices of eggplant and other fruits and vegetables fluctuate the most under the epidemic. This is related to the high price of eggplant itself and the imbalance of market supply (eggplant consumption in many parts of China depends on other provinces for supply). Among them, prices in Shanghai and Inner Mongolia increased by 3.40 RMB/kg and 3.05 RMB/kg respectively, with an increase of more than 3 yuan, making these the regions experiencing the largest increase. Affected by its economic structure, about 60% of all vegetables in Shanghai are supplied from elsewhere [22], but in February 2020, COVID-19 caused great difficulties in trans-provincial and trans-regional vegetable transportation. In Inner Mongolia under the influence of its predominant plateau landform, due to the lack of transportation routes, the traffic controls implemented to curb the epidemic impede haulage of such products.

### Intelligent prediction of vegetable prices based on machine learning

#### (1) Establishment of a ARIMA model

Firstly, the vegetable-price data are pre-processed; the “Remove duplicates” function in MS-Excel^®^ software is used to delete duplicate data records. For the missing values, there are many missing dates in the price records of the vegetable network data, to preserve the overall characteristics of the data, the missing dates of the price data are removed in the present research. In addition, given the fitness of time and price data in the time series model, the arithmetic mean of the prices in each market in the country is selected herein as the unique price corresponding to each date in the ARIMA forecasting model.

The following ARIMA forecasting model is established using cabbage data as an example.

Step 1: Raw series test

**Fig 12:**
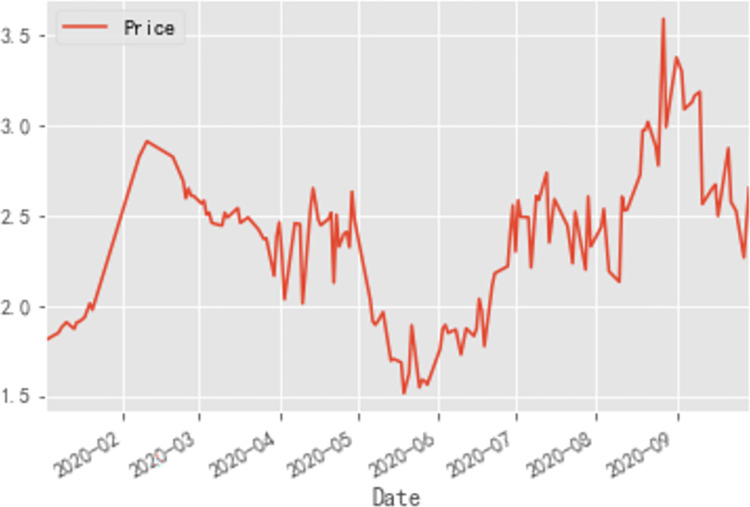
Time series of cabbage prices from January to September 2020.

**Fig 13:**
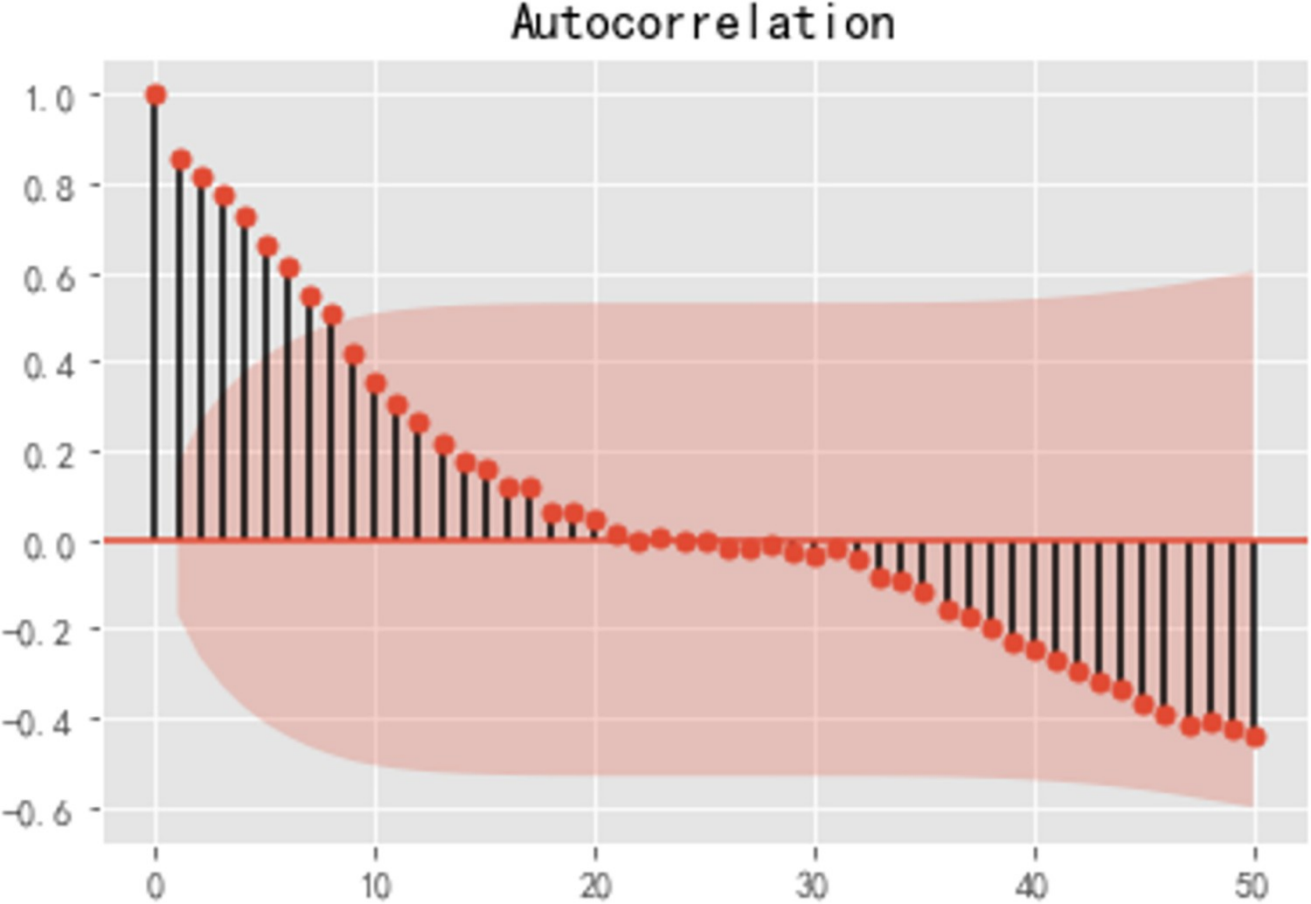
Cabbage price autocorrelation.

After importing the tool library and data, the Jupyter Notebook, a Python editor, is adopted to plot the cabbage price time series and the cabbage price autocorrelation graph. As shown in Fig 12, the overall time series of cabbage market prices fluctuates slightly. As illustrated in Fig 13, the autocorrelation coefficient for wholesale cabbage prices does not converge to zero, which implied that the series has a strong long-term correlation, suggesting that the series has some regularity and can be fitted and forecasted using the ARIMA model.

Next, the augmented Dickey-Fuller (ADF) test, a statistical test, is undertaken to test the smoothness of the original series of cabbage prices through python programming. The ADF test results for the raw series are: (-2.0112059061223695, 0.2816814175345441, 2, 134, {‘1%’: -3.480118600110386, ‘5%’: -2.8833618426136196, ‘10%’: - 2.578407034974382}, -51.368766678821004). From the test results, the second value—*p*-value—is shown to be greater than the significant level of 0.05 and therefore the original hypothesis is accepted, *i*.*e*. the original series is a non-stationary series and therefore a difference operation has to be applied to the series to make the series stationary before the model can be established.

Step 2: Differential operations

**Fig 14:**
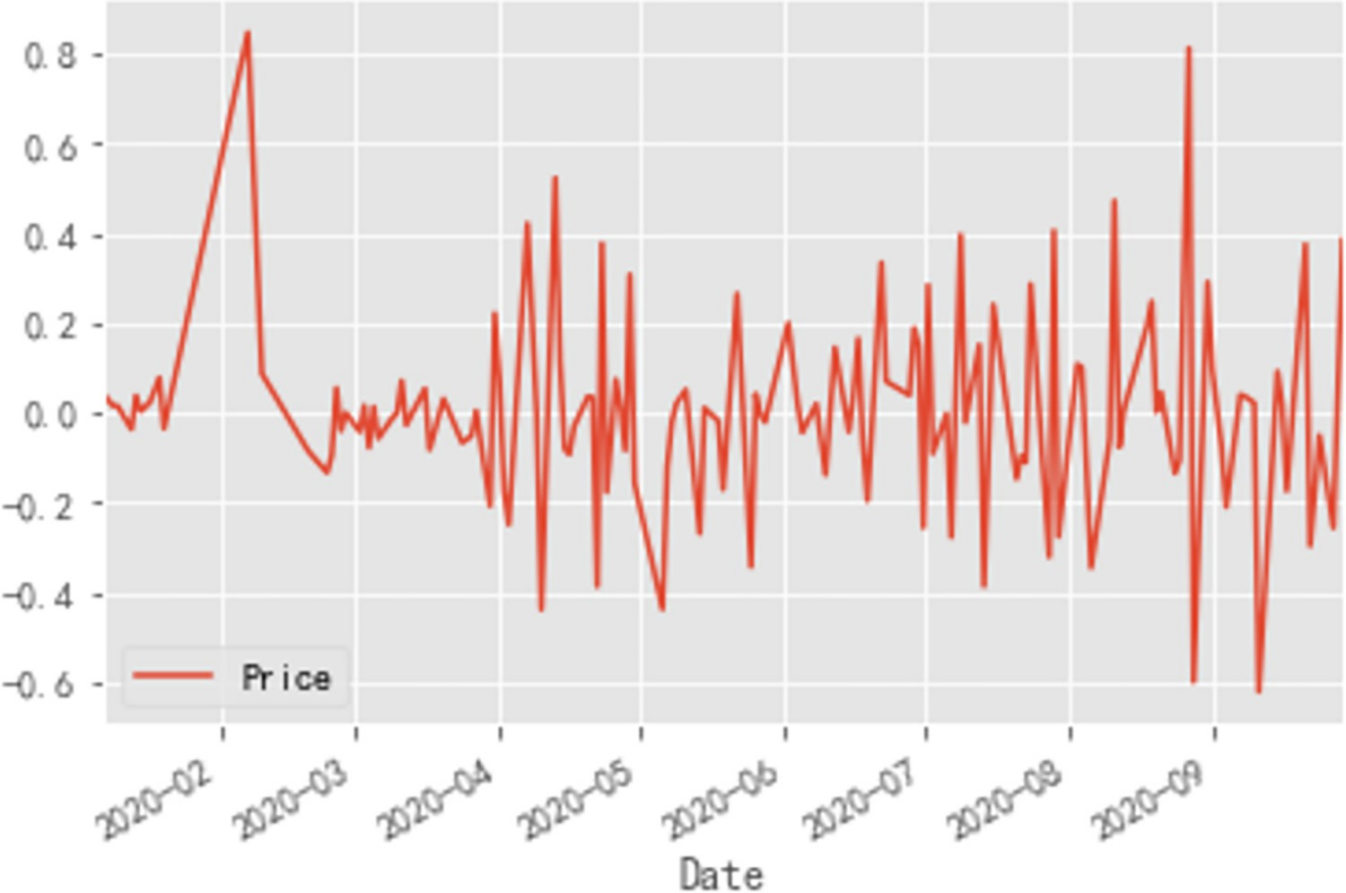
First-order difference time series diagram of cabbage prices.

First-order differencing is first performed and the time-series is plotted thereafter. As shown in Fig 14, the first-order difference series is smoother than the original series, but still fluctuates somewhat around the mean value.

A statistical test is employed to test the smoothness of the first-order difference series. The ADF test results for the first-order difference series are: (-11.362645236864235, 9.391004669119467e-21, 1, 134, {‘1%’:-3.480118600110386, ‘5%’:-2.8833618426136196, ‘10%’: - 2.578407034974382}, -49.535939206717046). From the test results, the *p*-value is found to be greater than the significant level of 0.05, so the original hypothesis is accepted, *i*.*e*. the original first-order difference series is a non-stationary series, so the difference operation is continued. Second-order differencing is conducted on the original series and the second-order differencing time series is plotted.

**Fig 15:**
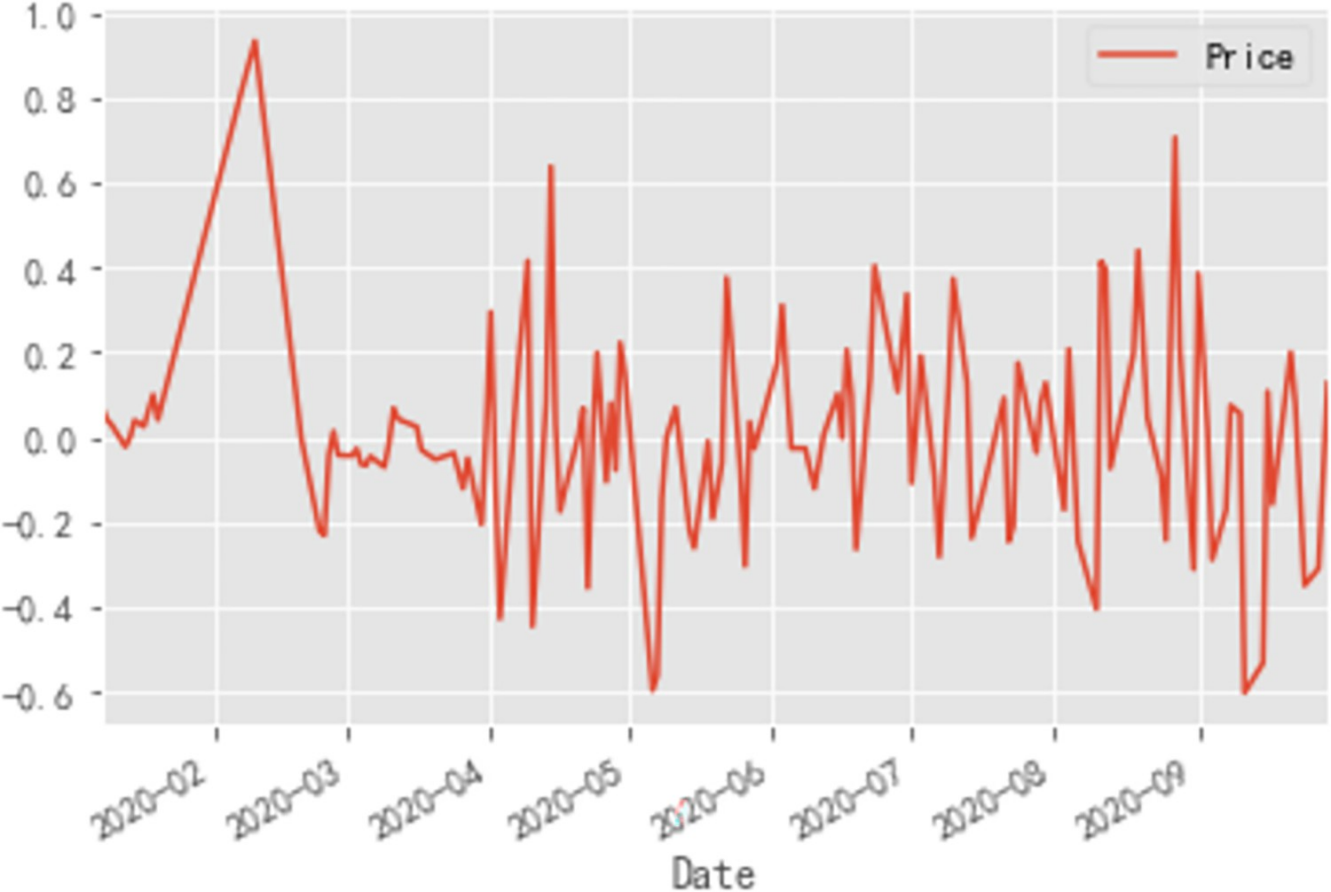
Second-order difference time series diagram of cabbage prices.

As shown in Fig 15, the second-order difference time series plot observation still fluctuates around the mean and there is no significant improvement in terms of smoothing from the image when compared to the original time series plot, but for more accurate smoothness testing, the same ADF test is used for the second-order difference sequence for statistical testing. Based on the results of the Python run, the ADF test for the second-order difference sequence is: (-3.468618670414781, 0.008830571318220638, 6, 128, {‘1%’: -3.4825006939887997, ‘5%’: -2.884397984161377, ‘10% ‘: -2.578960197753906}, -41.392014218569955). From the test results, the second value—the p-value is shown to be much smaller than the significant level of 0.05, so the original hypothesis is rejected, i.e. the second-order difference sequence is a smooth series, to this point, the second-order difference sequence can pass the smoothness test.

The white noise test is performed on the second-order difference sequence and the results of the white noise test for the second-order difference sequence are: (array(5.03943476), array(0.02477657)). The *p*-value is less than the significant level of 0.05, so the original hypothesis is rejected, i.e. the second-order difference sequence is non-white noise series and passes the white noise test, then the ARIMA model can be fitted. Therefore the final second-order difference series is taken, *d* = 2.

Step 3: Proposed merger to establish an ARIMA model

On the basis of determining *d* = 2 in the ARIMA(*p*,*d*,*q*) model, the *p* and *q*-values of the ARIMA model are determined by the Akaike Information Criterion (AIC) and Bayesian Information Criterion (BIC) methods. The input code defines a matrix and then establishes a loop to fit the ARIMA model continuously by combining different values of *p* and *q*. After running the program, the individual AIC or BIC values populate the matrix, taking the minimum value of each of the two matrices. In the cabbage price model, the final AIC value (-32.667081) and BIC value (-18.317173) are obtained. Comparing the values obtained by the two methods, the AIC value is found to be smaller, so the values of *p* and *q* obtained by the AIC method are used, *i*.*e*. *p* = 2 and *q* = 4 are taken, so the cabbage price model ARIMA(2,2,4) is constructed.

**Fig 16:**
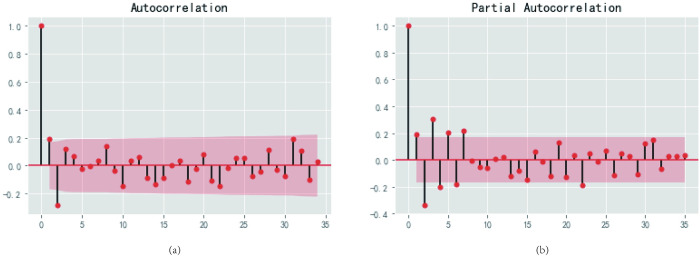
Residuals autocorrelation (a) and partial autocorrelation (b).

Residual tests are conducted on the model and the residual autocorrelation and partial autocorrelation are plotted, as shown in Fig 16. From the autocorrelation and partial autocorrelation plots of the residuals, it can be seen that the correlation coefficients are both small and therefore the correlation is not high, *i*.*e*. the difference in this ARIMA model is random.

**Fig 17:**
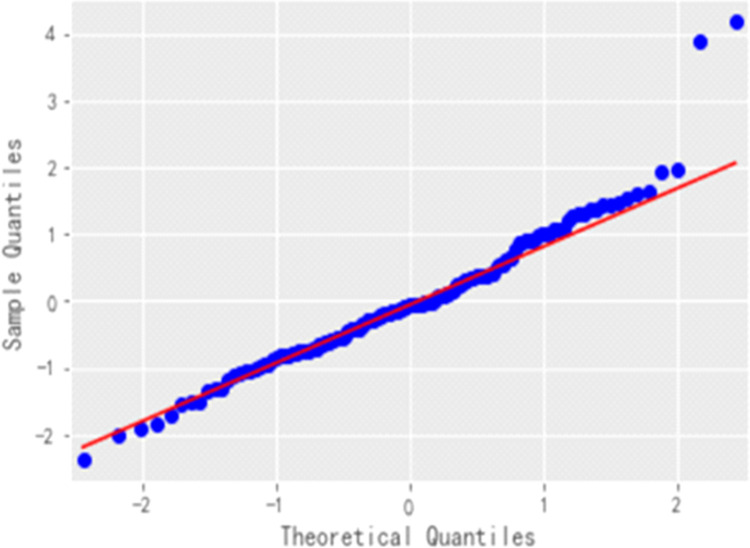
Residuals QQ plot.

The QQ plot is plotted using Python programming (Fig 17): the QQ plot is quasi-linear, *i*.*e*. the residuals obey a normal distribution with zero mean and constant variance, again indicating that the residuals are a purely random series. Once the residuals have passed the white noise test, it means that the establishment of the ARIMA model of cabbage prices is complete and the fitted ARIMA(2,2,4) model can then be adopted to forecast future price trends.

Step 4: ARIMA model prediction

**Fig 18:**
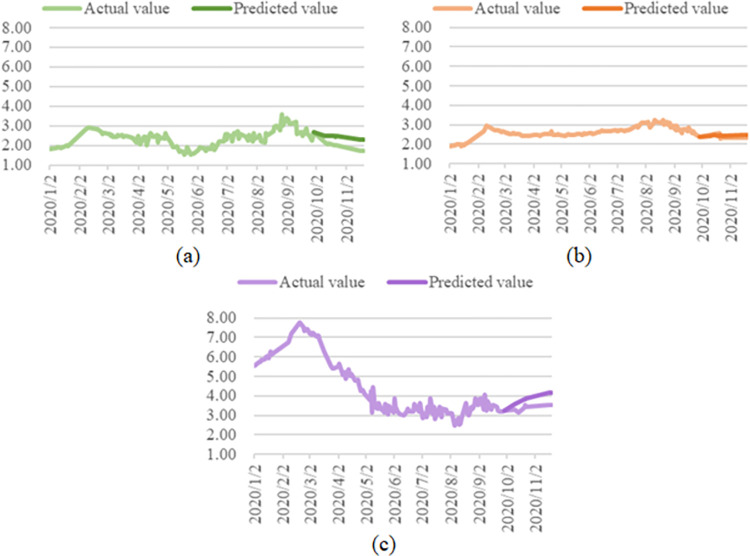
Average price forecast for cabbage (a), carrot (b), and eggplant (c).

Herein, using the average price series from January to September in 2020, the models ARIMA(2,2,4), ARIMA(7,0,3), and ARIMA(1,0,0) are built for the price data of cabbage, carrot, and eggplant respectively, and the vegetable prices from October 1 to November 19, 2020 are predicted by each model, and the results are plotted according to the prediction including the actual values and predicted values (Fig 18).

The average prices of cabbage, carrot, and eggplant in 2020 all showed an overall trend of first increasing, decreasing, then increasing. Due to the impact of the epidemic outbreak on the vegetable market, the vegetable market was in a tight balance at the beginning of the year, *i*.*e*. the supply and demand of vegetables were largely balanced in the short term, but there was not much remaining stock, thus preventing adequate supply in the vegetable market in the long term. As the impact of the COVID-19 pandemic on the vegetable market gradually waned, the state of supply and demand in the vegetable market gradually changed from a state of tight equilibrium to a state of sufficient supply. As shown in Fig 13, the forecast results from the ARIMA model indicate that the price forecast values do not differ much from the actual values, the forecast trend is generally consistent with the actual trend, and the price difference is controlled to within 1 RMB/kg. Vegetable producers should make corresponding adjustments to their planting plans for the second half of the year according to the real-time changes in the vegetable market, and try to refer to the intelligent price trend prediction results to minimize the impact of changes in the market prices of various vegetables on their own earnings.

#### (2) Model evaluation

**Fig 19:**
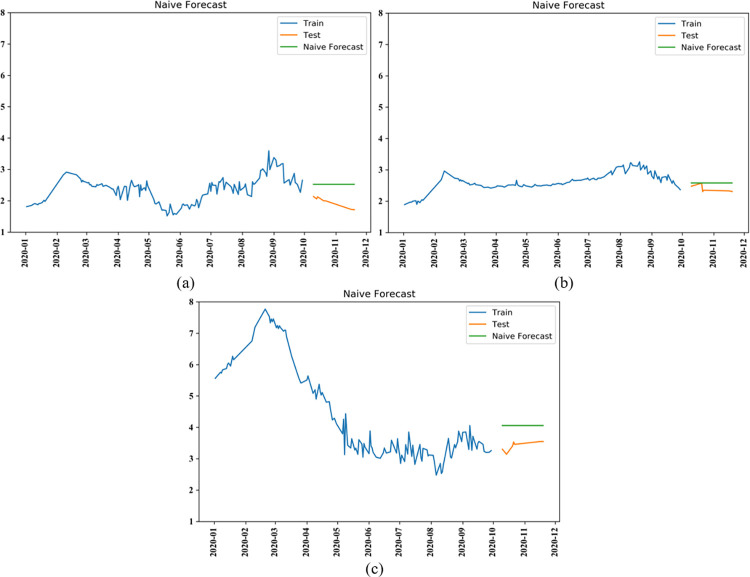
Naive forecast for price of cabbage (a), carrot (b), and eggplant (c).

In order to verify the effectiveness of ARIMA model, this paper compare it with naive forecast model. Similarly, the results of naive forecast using Python are shown in Fig 19. Where the green line is the predicted value and the orange line is the actual value. It can be seen that the graphs obtained from the naive forecast are all horizontal straight lines, which can not well simulate the trend of future vegetable prices like the ARIMA prediction curve.

Then, based on the prediction results of ARIMA model mentioned above, the prediction effects of the two models are compared and analyzed. Four commonly used forecasting model evaluation indicators: Mean Square Error (MSE), Root Mean Square Error (RMSE), Mean Absolute Error (MAE), and Mean Absolute Percentage Error (MAPE) are selected to evaluate the price forecasting results of cabbage, carrot, and eggplant, respectively (Table 1).

**Table 1:** Effectiveness of average vegetable price forecasts.

		MSE	RMSE	MAE	MAPE (%)
Cabbage	ARIMA	0.223	0.472	0.466	24.188
	Naive	0.335	0.579	0.558	29.213
Carrots	ARIMA	0.014	0.118	0.106	4.410
	Naive	0.040	0.199	0.170	7.240
Eggplant	ARIMA	0.217	0.465	0.447	13.078
	Naive	0.426	0.653	0.638	18.869

The closer these four parameters are to zero, the better the model predictions, and when the predicted value is exactly the same as the true value, it is a perfect model. The RMSE and the MAPE are often used as key indicators for judging forecasting models. The RMSE is employed to measure the extent to which the forecast deviates from the true value, *i*.*e*. the degree of dispersion in the sequence of forecast values. As the RMSE is more sensitive to error values, it is a good indicator of prediction accuracy and is often used as a criterion to estimate the accuracy of a model. The MAPE is used to determine the predictive effectiveness of a model—the magnitude thereof is proportional to the difference between the true and predicted values—when the MAPE exceeds 100%, it means that the model is of poor quality.

As can be seen from Table 1, the results clearly show that the ARIMA model used in this paper has better prediction effects than benchmark. Compared with the naive prediction model, the results predicted by ARIMA model show that the MSE, RMSE, and MAE of the models for the three vegetables were all more closer to zero, and the MAPE was much less than 100%. Comparing the ARIMA models among the price of three vegetables, the carrot price model provided the best prediction, with an absolute percentage error within 5% and a model fit accuracy of level 1, which better simulates the true value. Considering the impact of the COVID-19 pandemic on the vegetable market in 2020, a certain degree of error in the forecast of cabbage and eggplant prices is acceptable.

In summary, vegetable prices are influenced by a combination of multiple factors in all parts of the supply chain, and future changes of various factors are unpredictable. Therefore, considering the high uncertainty of vegetable prices, when vegetable producers make production and planting plans, compared with the naive forecast, the price forecast trend line that is more close to the actual value, namely Arima forecast curve, should be selected as the reference, help producers decide to increase, reduce or keep the output unchanged, and reduce the production risk of vegetable which with perishable characteristics.

## Discussion

**Fig 20:**
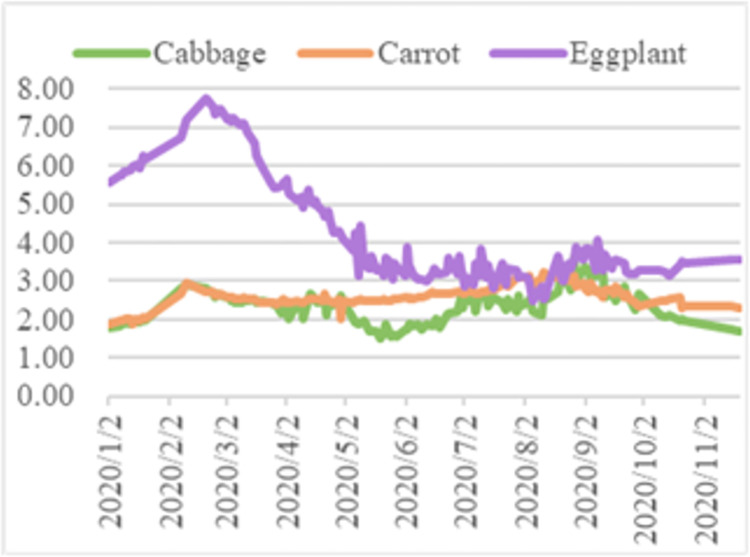
Average wholesale market price trends in 2020.

Taken together, the average wholesale market price trends for cabbage, carrots, and eggplants in 2020 are illustrated in Fig 20. Combining the results of the big data analysis of the vegetable price series with the forecasting results of the machine learning model, how the vegetable price series data affect the forecasting results of the ARIMA model is studied.

In summary, the price of eggplant was relatively volatile and on the whole showed a clearly downward trend, while the prices of cabbage and carrots exhibited less volatility. All three vegetables saw a peak in February and March, before prices began to fall and then peak again in September, so the price trends for all three vegetables were broadly similar, but the magnitude and specific values varied.

Observation of the data allowed us to explore the causes of variations therein:

1. The lowest value of cabbage prices was 1.52 RMB/kg on May 19, 2020, indicating that the epidemic prevention measures implemented by the State after the outbreak reached their optimal effect at the tail end of the epidemic interruption phase. The highest value for cabbage prices was 3.59 RMB/kg on August 27, 2020, when the epidemic was largely under control, so the peak was mainly a result of the summer impact on the vegetable production chain. The result of prediction model for cabbage, a perishable vegetable, was relatively poor due to the impact of the epidemic and seasonal effects around the year, respectively, and the strong disturbance in the prediction model for cabbage.
2. The highest value for carrot prices was 3.25 RMB/kg, occurring during the normalization phase of the epidemic prevention, indicating that the most influential factor on the peak carrot price was the seasonal factor. The lowest value of carrot price was 1.89 RMB/kg, which occurred at the beginning of the outbreak phase when the impact of the epidemic on vegetable prices had yet to be felt, and therefore the trough was mainly influenced by supply and demand, *i*.*e*. carrots being a seasonal vegetable therefore had a lower price when in season. In addition, carrot prices had the lowest peak-to-valley differential, indicating that root vegetables were the least affected by seasonal and epidemic factors, and therefore carrot prices were relatively less volatile and thus the best predictive model was found for carrot prices.
3. The highest value of eggplant price was 7.77 RMB/kg, which occurred at the beginning of the epidemic mitigation phase, mainly due to the peak price of eggplant as a result of soaring prices during the Chinese New Year, the impact of the epidemic and the counter-seasonal nature of eggplant in winter. The lowest value for eggplant was 2.47 RMB/kg, which occurred during the normalization phase of the epidemic and was mainly influenced by commodity-value factors. Eggplant, as a perishable vegetable was more perishable in summer, hence the low quality and low price of eggplant in summer appeared. In addition, the peak of eggplant prices was more than three times its minimum value, making it the vegetable with the largest extreme price difference. The large fluctuations indicate the unstable nature of the eggplant price series, so to establish an ARIMA model, the series must be different but its native fluctuation pattern still affects the model, resulting in relatively poor predictions for eggplant.

In summary, the disturbance in the price forecasting process is stronger for perishable vegetables than for non-perishable vegetables, and the data-driven ARIMA-based model is better for non-perishable vegetables, *i*.*e*. the ARIMA model is better suited to forecasting smoother series. As shown in Fig 14, comparing the price curves of the three vegetables, the price curve of eggplants was the most volatile. As shown in Table 1, the predicted price of eggplant was closer to the price level at the beginning of the year after the 2020 epidemic had largely stabilized, giving a more favorable prediction. In contrast, the predicted value was closer to the pre-epidemic price level than the actual value, indicating that the price of eggplant was more influenced by the epidemic. In particular, as shown in Fig 5, the most volatile phase of the eggplant price was the epidemic interruption phase, when it fell from 6.36 RMB/kg to 3.14 RMB/kg, a drop of over 50%. This finding indicates that during the epidemic interruption phase, both government and relevant enterprises should pay close attention to the movement of eggplant prices and implement macro-regulation: government and enterprises should collect price data of vegetables timeously and establish an early-warning system for price fluctuations through artificial intelligence, so as to alert vegetable farmers and consumers in advance to take countermeasures and mitigate future price fluctuations, thereby promoting the stable operation of the vegetable market.

## Conclusion

### Summary of vegetable price data analysis and predictions

Price is the most sensitive reflector in the market. Forecasting the trend of vegetable prices in advance is directly related to the development of the vegetable industry as well as the livelihood of farmers and residents. In real-life applications, the use of ARIMA models for forecasting vegetable prices, together with the specific market environment, can help government departments to regulate the supply and demand of the vegetable industry, as well as provide scientific guidance and support for farmers, agricultural bases and enterprises to optimize vegetable production, to promote the high-quality development of the vegetable industry. Vegetable price series can be analyzed and predicted based on big data analysis, mining and intelligent forecasting during the epidemic and the conclusions are drawn as follows:

1. Vegetable price fluctuations in 2020 were mainly affected by the epidemic factor and seasonal factors. During the epidemic, the demand for vegetables from both urban and rural residents increased significantly, further exacerbating the imbalance in matching supply and demand for vegetables and leading to fluctuations in vegetable market prices.
2. The forecasting accuracy and stability of any forecasting model are negatively correlated with the forecasting interval. In short and medium-term forecasting studies, more emphasis is placed on the fit of the forecast results themselves to the true values, while long-term forecasting tends to focus more on describing the future development trend in the forecast object. In comparison, the ARIMA model used in the present research is suitable for short-term forecasting, while the effectiveness of long-term forecasting remains to be verified [23, 24]. In practical applications, the prediction interval should be flexibly adjusted according to the research needs to improve the accuracy and reliability of the prediction results, and data-driven long-term forecasting analysis of vegetable prices is also one of the main directions of our future research.
3. According to the prediction results, the ARIMA model used in this paper is better than the benchmark model, and puts forward corresponding production suggestions based on its prediction trend: for leafy vegetables such as cabbage, where the forecast price trend is slowly declining, producers should switch production plans in time or adopt appropriate marketing strategies to reduce the risk of future economic losses; if the forecast vegetable price trend is similar to that of root vegetables in the data, which is stable and does not fluctuate much, then producers should maintain their production: if the price trend for vegetables is similar to that for eggplant vegetables in the data, which is on the rise, then producers should increase production—by making full use of the growing season and sunlight exposure to increase production, or simply expanding the area under vegetable cultivation, but they also need to know the extent of the increase so that the supply of vegetables does not outstrip demand and lead to a glut. The extent of any increase in production needs to be managed so that the supply of vegetables does not outstrip demand and lead to a backlog of stocks.

### Problems and optimization of the vegetable supply chain

After analyzing the influencing factors of vegetable price data from horizontal and vertical perspectives and price-intelligence prediction, it is concluded that there are mainly problems in the vegetable supply chain caused by COVID-19 in 2020:

1. In the production link, vegetable production is affected by climate and other environmental factors. In addition, there are significant differences in vegetable planting structure across China, which leads to the dispersed nature of vegetable production areas. However, after the outbreak, the prices of vegetables in different regions varied greatly (Fig 11). This shows that the vegetables produced by many scattered farmers all over the country failed to achieve benign resource integration. Therefore, the phenomenon of vegetable price fluctuation caused by the spread of the epidemic is very common. Under the great demand caused by the outbreak of the epidemic, these fragile vegetable production entities lack emergency capacity and find it difficult to maintain the stable supply of vegetables that may have otherwise allowed more stable prices throughout the country.
2. In the circulation link, in the face of the sudden epidemic, the emergency response capacity of the vegetable supply chain is insufficient, and the vulnerability of its supply system is exposed. Especially in remote areas such as Gansu and Inner Mongolia, there are few transportation lines. After the outbreak of the epidemic, the problems in the circulation link are becoming increasingly prominent. The closure of cities and roads under the implemented epidemic prevention work have caused difficulties to the transportation of vegetables, especially the long-distance transportation of vegetables across provinces and regions. Poor circulation has led to a steep rise in the price of perishable leafy vegetables (such as Chinese cabbage). The dispersion of vegetable production and market leads to obvious price fluctuation and obvious price increases. As shown in Fig 7, compared with the epidemic-free period, the price rise of cabbage in the northern region with scattered vegetable markets in 2020 is more obvious than that in the south.
3. In the sales link, the information pertaining to vegetable production and marketing places is asymmetric, and many farmers have few channels and little ability to obtain vegetable market information timeously. Under the outbreak of the epidemic, the information of all links in the vegetable supply chain does not form a complete closed loop, resulting in unsalable vegetables in place of origin, while vegetables being difficult to find on place of sale. As shown in Fig 11, since most vegetables in Shanghai are transported from elsewhere, blockages therein during the epidemic, resulted in a shortage of eggplant supply and a sharp rise in prices. In 2020, the epidemic control measures closed the wholesale market and farmers’ market. The low degree of information integration in the vegetable supply chain resulted in a huge waste of resources and economic losses.

“Rome was not built in a day”. such problems were originally the bottleneck to development of China’s vegetable supply chain, and became more prominent under the sudden COVID-19 epidemic.

1. In the production link, due to the comprehensive influence of climate, soil, terrain, and other factors, the vegetable planting structure in China varies greatly. For example, vegetables in most of the areas in the north of the Qinling Huaihe River line are planted in dry land, while the areas south of the Qinling Huaihe River line have more vegetable planting categories due to the greater amount of water available. For example, the price of wet cabbage is more stable to the south of Qinhuai River (Fig 7); the price of eggplant (given the risk of waterlogging and crop-loss) is more stable to the south of Qinhuai River (Fig 11). Therefore, government should make use of macro-control, giving full play to the agricultural advantages of various regions, and gradually integrate the scattered farmers, production bases, agricultural cooperatives, and other vegetable business entities from point to area, so as to facilitate the rational allocation of resources over a more macroscopic scope in the national vegetable supply chain and achieve the dynamic balance of vegetable prices in all parts of China.
2. In the circulation link, government can support the development of the vegetable supply chain through national policies, integrate national logistics resources, and provide scientific and efficient logistics infrastructure and logistics channels for vegetable circulation. To reduce the deterioration rate of vegetables in the circulation link (especially leafy vegetables), we should increase the investment in cold chain logistics facilities and plan to establish large-scale cold storage throughout the country, so as to reduce the loss of vegetables caused by temperature deterioration in the circulation link. Through the government’s macro-control, we aim to realize “whole process cold chain” management of the vegetable supply chain, and actively build a cold chain distribution and transfer center for vegetables across provinces and regions, so as to facilitate the efficient circulation of vegetables throughout the country. While improving the freshness of vegetables, the circulation cost of vegetables also need to be controlled. Different cold chain levels are established according to the risk of perishing of different vegetables and a cold chain is constructed according to local conditions, and the cooperation among the main bodies of the national vegetable supply chain is strengthened through industry leadership, thus forming a “vegetable circulation ecosystem” with shared cooperation throughout. In the post-epidemic era, the high-level cold chain logistics system of vegetable supply chain cannot only improve the freshness of vegetables under normal circumstances, but also prolong the waiting time for deliveries of vegetables in case of an epidemic outbreak, so as to lay a time-basis for long-distance transportation of vegetables.
3. In the sales link, with the help of a fresh e-commerce platform to convey vegetable sales, the cost of intermediate links and the price of vegetables can be reduced. The fresh e-commerce platform is conducive to collecting dynamic information in the vegetable supply chain in real time, publishing and sharing it timeously, and helping vegetable producers and sellers grasp the dynamics of the vegetable market in real time. Meanwhile, considering the differences of information channels that people of different ages and educational backgrounds pay attention to when identifying information [25], training in information channel communication and sharing should be condcuted to improve their familiarity with the fresh food platform. Through the vegetable information on the e-commerce platform, vegetable producers can better adjust the vegetable production and planting plan, while vegetable sellers can also analyze their own sales situation according to the current vegetable market conditions, and timeously achieve sales goal by adjusting vegetable pricing, sales methods, and other means. In the post-epidemic era, the fresh e-commerce platform can strengthen the tightness of vegetable production and marketing, and avoid the imbalance of vegetable supply caused by asymmetric production and marketing information. When an epidemic breaks out, producers, sellers, consumers, and government can be fully cognizant of the vegetable production and inventory status all over the country in real time, allowing broader vegetable distribution and circulation.

### Prospects

In addition, there are a number of areas that can be improved.

1. The ARIMA model established herein through observing, describing and studying the change characteristics of time series, looks for the development law of data, predicts its future trend and constructs the corresponding time series model. In time, the environment and context in which the data are located will also change, therefore, according to the results of the big data analysis, adding seasonal factors to the model can improve price forecasting results. At the same time, how to adjust ARIMA model and apply it to long-term prediction is also a direction worth studying.
2. An early-warning mechanism for vegetable market prices in China can be improved based on seasonal changes and price forecasting models: we should aim to integrate real-time trading volume and trading price data from wholesale markets, fresh produce e-commerce enterprises and futures markets, introduce various influencing factors as price warning factors, establish a monitoring and statistical system from the field to the table, strengthen vegetable data and model analysis, and serve national macro-control decisions.
